# Platelet-to-albumin ratio and radiation-induced lymphopenia—prognostic biomarker for carcinoma esophagus

**DOI:** 10.1186/s43046-024-00208-4

**Published:** 2024-02-05

**Authors:** Adrija Ghosh, Abhilash Dagar, Ram Pukar Bharat, Jaswin Raj, Dyuti Shah, Jyoti Sharma, Akash Kumar, Pritee A. Patil, Aman Sharma, Dayanand Sharma, Supriya Mallick

**Affiliations:** 1 Department of Radiation Oncology, All India Institute of Medical Sciences, New Delhi, India; 2 Department of Surgical Oncology, All India Institute of Medical Sciences, New Delhi, India; 3 Department of Medical Oncology, All India Institute of Medical Sciences, New Delhi, India

**Keywords:** Esophageal cancer, Platelet-to-albumin ratio, Prognosis, Neutrophil-to-lymphocyte ratio, Platelet-to-lymphocyte ratio

## Abstract

**Background:**

Esophageal cancer has a poor survival outcome with 5-year OS at 16.7% despite treatment. Some inflammation-based prognostic indicators like the neutrophil-to-lymphocyte ratio (NLR) and platelet-to-lymphocyte ratio (PLR) have been previously studied as potential biomarker for predicting outcome in esophageal cancer. Recently, platelet-to-albumin ratio (PAR) has been reported as a promising prognostic factor in gastrointestinal malignancies.

**Methods:**

We performed a retrospective analysis of prospectively treated patients of carcinoma esophagus to evaluate the prognostic significance of inflammation-based prognostic indicators—neutrophil-to-lymphocyte ratio (NLR), platelet-to-lymphocyte ratio (PLR), and a composite inflammation-nutrition index: platelet-to-albumin ratio (PAR) in esophageal cancer. Based on previous studies, the optimal cut-off value of PAR was kept at 5.7 × 10^9, and 2.62 for NLR.

**Results:**

A total of 71 patients of locally advanced esophageal cancer treated between 2019 and 2022, with either neoadjuvant or definitive chemoradiotherapy, were included. Median follow-up time was 19 months [range: 7–44 months]. Median OS and PFS in our study cohort were 11.3 months [range: 7–23 months] and 7.8 months [range: 3–17 months], respectively. In univariate analysis, lower PAR was found to be significantly correlated with shorter survival time (HR = 2.41; 1.3–4.76; *p* = 0.047). There was no association found between the OS and the NLR [HR = 1.09; 0.95–1.26; *p* = 0.222]. Univariate and multivariate linear and logistic regressions found no association between V15, V10, V5, or V2 of spleen and nadir lymphocyte count or between Dmax or Dmean and nadir lymphocyte counts.

**Conclusion:**

Present analysis found a trend toward an inverse association between PAR and OS. PAR, in the not-so-distant future, may evolve as a novel, convenient, and inexpensive prognostic indicator in esophageal cancer.

## Introduction

Burden of esophageal cancer worldwide makes it the ninth most common cancer and the sixth leading cause of cancer-related mortality worldwide [1, 2]. Treatment of esophageal cancer depends on the stage, nodal involvement, and tumor location. In resectable esophageal cancer, standard of care is pre-operative concurrent chemoradiotherapy followed by resection [3]. In others, concurrent chemoradiotherapy followed by consolidation chemotherapy forms the backbone of the treatment [4]. Despite the advances in all available modalities of treatment, oncologic outcome of esophageal cancer remains dismal with 5-year survival rates ranging from 20 to 30% only [5, 6]. In light of these precarious statistical figures, it becomes all the more necessary to identify various prognostic markers that may help us in further treatment escalation.

Studies have shown that baseline nutritional status, cancer-related inflammation, the immune system, and thrombosis affect oncological outcomes across various solid tumors including stomach, lung, and prostate cancer, in terms of carcinogenesis, proliferation, progression, and metastasis [7–12]. Thus, assessing a patient’s pre-treatment nutritional and inflammation status becomes imperative in order to attempt to bring about a positive survival impact. However, screening tools for evaluating the perioperative nutrition and inflammation status in esophageal cancer patients are currently limited. Some inflammation-based prognostic indicators like the neutrophil-to-lymphocyte ratio (NLR) and platelet-to-lymphocyte ratio (PLR) have been previously studied in esophageal cancer [13]. Recently, platelet-to-albumin ratio (PAR) has been reported as a promising prognostic factor in gastrointestinal malignancies [14–16]. Previous reports have shown that platelets are a marker of systemic inflammation status, and albumin is one of the most important markers of nutritional status which might make PAR a composite practical low-cost surrogate marker of both the nutritional status and systemic inflammation status. Some studies also suggest that unintended splenic irradiation in lower thoracic esophageal cancer correlates to the severity of lymphopenia and, ultimately, a lower survival outcome [17–19].

We wanted to evaluate the prognostic significance of inflammation-based prognostic indicators—neutrophil-to-lymphocyte ratio (NLR), platelet-to-lymphocyte ratio (PLR), and a composite inflammation-nutrition index: platelet-to-albumin ratio (PAR) in esophageal cancer. We also wanted to identify if unintended spleen irradiation correlated to the nadir lymphocyte count. The protocol was approved by institute ethics committee.

## Materials and methods

### Patients

Data of patients with esophageal cancer who underwent chemoradiotherapy on curative lines between November 2019 and October 2022 were retrospectively reviewed. Eligible patients had histologically confirmed SCC of the esophagus; ECOG PS 0–2; and treated with definitive or neoadjuvant chemoradiotherapy. Patients with a history of previous malignancy or chronic/acute inflammatory diseases were not considered for analysis.

### Treatment protocol

All patients were staged according to the 8th AJCC TNM staging for esophageal cancer, with the aid of imaging techniques for assessment of the locoregional extent of the disease and to rule out distant metastases. A multidisciplinary board comprising of a radiation oncologist, a medical oncologist, a radiologist, a nuclear medicine physician, and a surgical oncologist finalized the treatment plan. Patients deemed resectable underwent neoadjuvant chemoradiotherapy to a dose of 41.4 Gy in 23 fractions treated five times a week over 4.5 weeks with concurrent weekly carboplatin-paclitaxel. If not eligible for surgery, patients underwent definitive chemoradiotherapy till 50.4 Gy in 25 fractions over 5 weeks with weekly 5FU-cisplatin followed by two cycles of consolidation chemotherapy. The patients deemed resectable by the MDT underwent Ivor Lewis esophagectomy or VATS assisted TTE.

#### Radiation planning

**Fig. 1:**
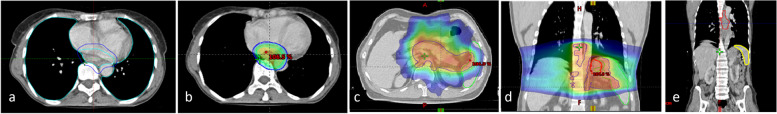
**a** Target delineation showing the CTV in cyan blue and PTV in royal blue, **b** target volume coverage showing the 95% dose color wash, **c** 50% dose color wash showing splenic dose distribution; **d** 10% dose color wash showing splenic dose distribution, **e** retrospective delineation of the spleen (yellow)

**Fig. 2:**
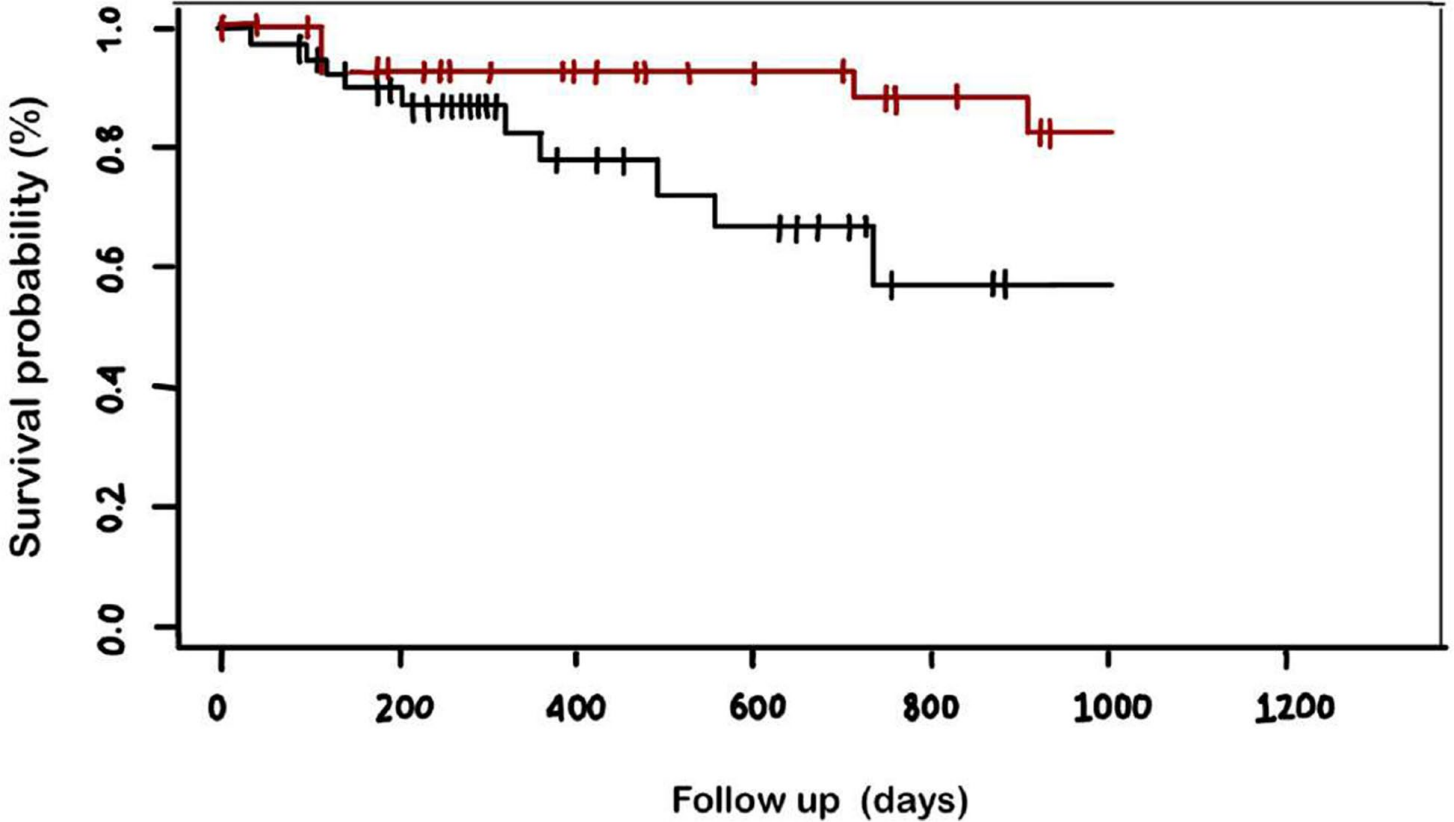
Kaplan–Meier survival curve showing the overall survival outcomes between the low and high PAR group. Black—high PAR group; red—low PAR group. HR = 2.41; 1.3–4.76; *p* = 0.047

Gross tumor volume (GTV) delineation for radiotherapy was done after co-registration of the PET-CT images with the planning CT images and correlation with the upper GI endoscopy findings. A 3-cm craniocaudal expansion along the esophageal mucosa and a 1-cm circumferential margin (anatomically constrained) were applied to form the CTV. Lymph nodes if seen were contoured separately; a 1-cm margin was given to form the nodal CTV. A 1-cm margin was applied to the CTV to form the planning target volume (PTV) (Fig. 1a). Radiotherapy was delivered by the IMRT (intensity-modulated radiation therapy) or the VMAT (volumetric modulated arc therapy) using the Eclipse version 15.5 treatment planning system (Varian Medical, Palo Alto, CA) (Fig. 1b). VMAT/IMRT was delivered with 6-MV Acuros-XB algorithm version 15.6.05 photon beams generated from the Varian TrueBeam SVC linear accelerator equipped with a 120-leaf Millenium multi-leaf collimator. Spleen was retrospectively contoured for all the patients and the spleen dosimetry was evaluated in terms of the mean spleen dose (Dmean), the maximum spleen dose (Dmax), V2, V5, V10, V15, and V20 (Fig. 2).

### Follow-up

Patients were reviewed weekly during the course of treatment for acute toxicities. After completion of treatment, follow-up examinations were conducted 1 month after finishing radiotherapy, and then every 3 months in the first year, every 6 months over the next 2 years, and once a year thereafter with physical examination, thoracic CT scanning, or 18FDG whole body PET-CT scan. Overall survival (OS) was defined as the period from treatment initiation to the date of last follow-up or death from any cause. Progression-free survival (PFS) was defined as the period from treatment initiation to the date of disease progression or death from any cause. Disease progression was evaluated by the standard Response Evaluation Criteria in Solid Tumors (RECIST criteria).

### Definition of the indices

The baseline hematological parameters, namely, the hemoglobin, neutrophil, lymphocyte, platelet counts, and serum albumin levels, were collected from the central database system of the hospital and the corresponding neutrophil–lymphocyte ratio (NLR) and platelet-albumin ratio (PAR) were calculated. The same was repeated for weekly laboratory values during the course of concurrent chemoradiotherapy and for values obtained 1 month after completion of the designated treatment. The nadir lymphocyte count during the course of radiotherapy was considered in every patient and it was graded according to the Common Terminology Criteria for Adverse Events version 4.0.

NLR and PAR were defined as NLR = absolute neutrophil count/absolute lymphocyte count and PAR = platelet counts/serum albumin level (g/L). Based on previous studies, the optimal cut-off value of PAR was kept at 5.7 × 10^9, and 2.62 for NLR [20]. The cohort of the patients selected was stratified into a low PAR (PAR < 5.7 × 10^9) and a high PAR group (PAR ≥ 5.7 × 10^9).

### Statistical analysis

All statistical analysis was performed with SPSS 26.0 (SPSS, Chicago, IL). Association between the PAR groups, the NLR, and clinicopathological characteristics was analyzed by the χ^2^ test. Survival curves for OS were plotted via the Kaplan–Meier method and compared by the log-rank test to assess the prognostic influence of the NLR and the PAR. Univariate and multivariate Cox analyses were conducted to identify the independent risk or prognostic factors. A *p* value < 0.05 was considered statistically significant. Spearman correlation coefficients were used to evaluate the associations between the spleen dose-volume parameters and the nadir lymphocyte count.

## Results

### Patient demography

Baseline clinicopathological characteristics of the patients eligible for the analysis have been presented in Table 1.

**Table 1:** Clinicopathological characteristics of the selected patient cohort

Characteristic	Patients (*n* = 71)
Age median (range)	57 years (29–75 years)
Gender	
Male	47 (66.19%)
Female	24 (33.8%)
ECOG PS	
0	25 (35.2%)
1	35 (49.2%)
2	11 (15.5%)
Location	
Cervical	2 (2.8%)
Upper thoracic	23 (32.3%)
Middle thoracic	27 (38.2%)
Lower thoracic	19 (26.70%)
Stage (8th AJCC)	
II	10 (14%)
III	33 (44.6%)
IV	28 (39.4%)
Intent	
Neoadjuvant CTRT	26 (36.6%)
Definitive CTRT	45 (63.4%)
RT technique	
IMRT	34 (47.8%)
VMAT	37 (52.2%)
PAR values	
High (PAR ≥ 5.7 × 10^9)	27 (38%)
Low (PAR < 5.7 × 10^9)	44 (62%)

*CTRT*, Chemoradiotherapy, *IMRT*, Intensity-modulated radiotherapy, *VMAT*, Volumetric modulated arc therapy, *PAR*, Platelet-to-albumin ratio

### Prognostic significance of PAR and NLR

**Fig. 3:**
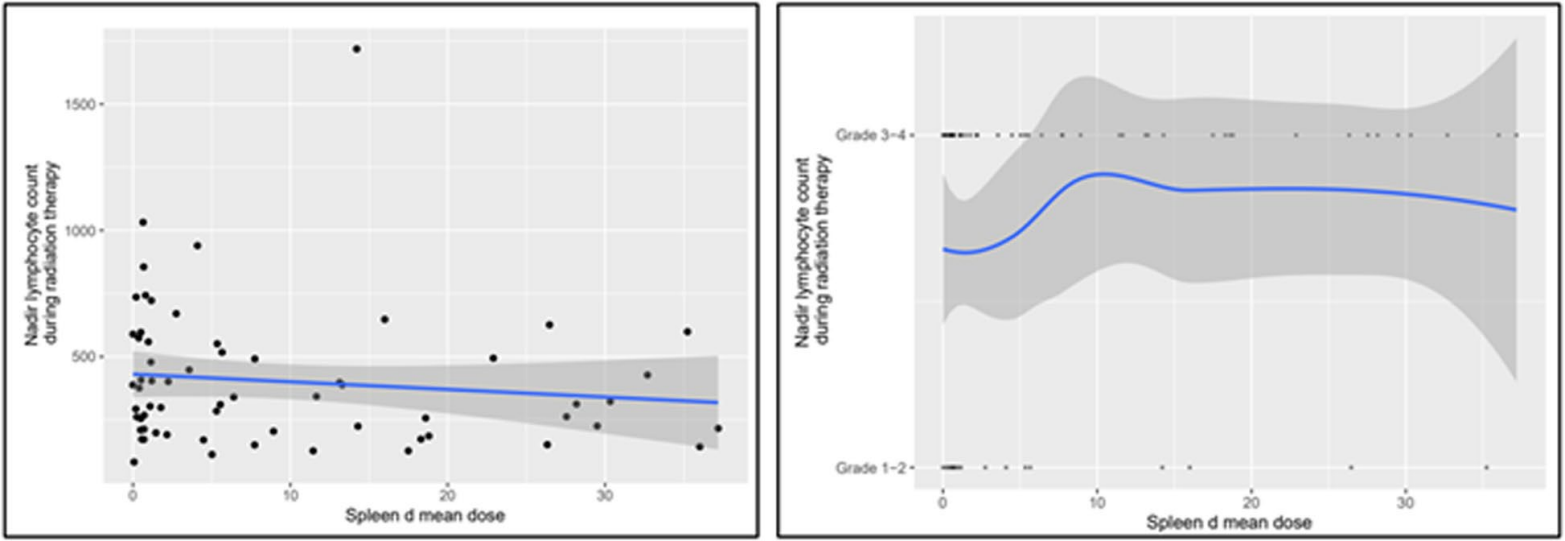
Correlations between spleen mean dose and nadir lymphocyte absolute count and nadir lymphopenia grade during radiotherapy in patients with esophageal cancer

**Fig. 4:**
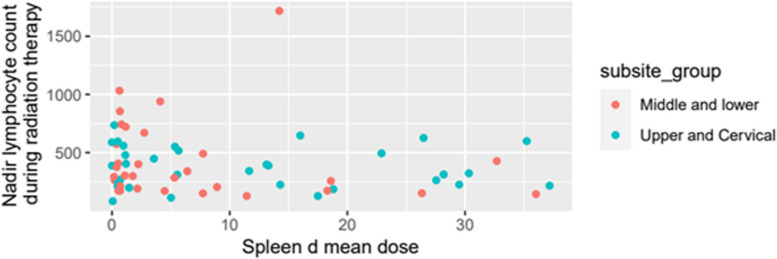
Correlations between spleen mean dose and nadir lymphocyte absolute count during radiotherapy in patients with esophageal cancer on subset analysis

Median follow-up time was 19 months [range: 7–44 months]. Median OS in the study cohort was 11.3 months [range: 7–23 months] while median PFS was 7.8 months [range: 3–17 months]. In Kaplan–Meier survival analysis, lower PAR significantly correlated with shorter survival time (HR = 2.41; 1.3–4.76; *p* = 0.047) (Fig. 3). One- and 2-year OS rates were 76.5% and 58.9%, respectively, in the high PAR group and 87.1% and 82.4%, respectively, in the low PAR group. Kaplan–Meier analysis showed no association found between survival and NLR [HR = 1.09; 0.95–1.26; *p* = 0.222]; nadir lymphocyte count [HR = 1.21; 0.87–1.36; *p* = 0.34]; platelet-lymphocyte ratio (PLR) [HR = 1.1; 0.98–1.21; *p* = 0.56]; or the serum albumin [HR = 0.99; 0.87–1.29; *p* = 0.78]. Univariate analyses showed that lymph node metastasis, T stage, TNM stage group, and PAR were predictive of overall survival (OS) (Table 2). Multivariate analyses were performed using the Cox proportional hazards model for these variables, which identified the TNM stage group (*p* = 0.005) and lymph node metastasis (*p* = 0.031) as predictors of OS. PAR lost its significant impact on OS on multivariate analysis (Table 3). Spearman correlation analysis revealed that V2, V5, V10, V15, V20, Dmax, and mean splenic dose did not significantly correlate with nadir lymphocyte count (*p* = 0.41, 0.61, 0.21, 0.09, 0.87, 0.58, 0.06, respectively) (Fig. 4). Subset analysis did not show any relation between these even for middle and lower esophagus subsites. Spleen Dmean was significantly more with VMAT than IMRT (*p* = 0.02). Out of the 71 patients audited, 45 patients (63.4%) were planned for definitive CTRT while the remaining underwent neoadjuvant chemoradiotherapy and were subsequently planned for surgery. However, only 15 (60%) of those planned for surgery did actually got operated. In survival analysis, lower PAR significantly correlated with shorter survival time in both groups individually (HR = 2.62; 1.7–4.7; *p* = 0.037) (HR = 2.42; 1.3–4.3; *p* = 0.043). However, other hematological markers analyzed did not show significant survival impact in either of the groups, nor did the survival among the two groups vary significantly.

**Table 2:** Univariate analysis of prognostic factors influencing survival rate

Characteristic	Cases	Median survival (months)	*p* value
Gender			0.112
Male	47	11	
Female	24	13	
Age (years)			0.082
≤ 60 years	42	12.6	
> 60 years	29	10.8	
Tumor location			0.127
Cervical	2	9.7	
Upper	23	12.4	
Middle	27	11.2	
Lower	19	11.8	
PET length			0.061
≤ 3 cm	23	13.5	
> 3 cm	48	10.7	
T stage			0.045
T3	43	15.4	
T4	28	9.8	
Lymph nodal status			0.023
N0	37	16.8	
N +	34	8.6	
TNM stage			0.003
II	10	14.5	
III	33	12.6	
IV	28	8.3	
Nadir lymphopenia			0.068
Grades 1–2	35	12.5	
Grades 3–4	36	10.6	
NLR			0.222
< 2.62	29	11.9	
≥ 2.62	42	9.9	
PAR			0.047
< 5.7 × 10^9	44	18.7	
≥ 5.7 × 10^9	27	10.5	
Serum albumin			0.78
≤ 2.5	39	9.6	
> 2.5	32	11.3	

*NLR*, Neutrophil-to-lymphocyte ratio, *PAR*, Platelet-to-albumin ratio

**Table 3:** Multivariate analysis of selected prognostic factors influencing survival rate

Characteristic	HR	*p* value
Lymph nodal status	1.46	0.031
T stage	1.23	0.058
TNM stage group	1.65	0.005
PAR values	1.29	0.067

## Discussion

Esophageal carcinoma is a highly aggressive malignancy which makes it imperative to identify various prognostic factors besides the TNM staging system, to screen high-risk patients who are more pronounced to experience or distant metastasis, and to implement aggressive early intervention in an attempt to improve the outcomes on a personalized basis. Recent studies suggest that cancer-related inflammation (CRI) is the seventh hallmark of cancer and presence of a smoldering inflammation in the tumor microenvironment contributes to proliferation and sustenance of malignant cells, metastasis, angiogenesis, and aversion of the host tumor-related immune response which ultimately lead to a resistance pattern toward systemic anticancer therapies [21].

Platelets, a critical coin in the phenomenon of hemostasis, are also now being linked to systemic inflammation and are gaining recognition as an immune modulatory cell [22, 23]. Moreover, platelet could shield peripheral circulating tumor cells and interfere with natural killer cells for recognition of tumor cells, which enhanced their metastatic potential [24]. Nutritional status is also an important aspect of the holistic management of cancer. Malnutrition and cachexia have been independently evaluated as poor clinical prognostic factors in patients with advanced cancer [25–27]. Earlier, albumin was reported to be a biomarker of malnutrition which significantly correlated with poor clinical outcomes in patients of esophageal cancer [28, 29]. Moreover, albumin synthesis is a negative inflammatory marker. Therefore, risk stratification based on inflammation-nutritional indicators is of great significance and will help the clinical physician to provide timely and effective nutritional intervention. That is how arose the novel practical surrogate marker known as the platelet-albumin ratio (PAR) which takes into account both the inflammatory and nutritional status of the patient. This marker can be easily calculated from a simple hemogram and a liver function test, which makes it very practical. Huang et al. showed that PAR could be an independent indicator of PFS and OS. Patients with a low pre-treatment PAR (< 5.7 × 109) had a significantly better prognosis in both PFS and OS than those with a high pre-treatment PAR (≥ 5.7 × 10^9^) [20]. Another study found a marginally significant difference in the post-operative surgical complications and long-term oncological outcomes between the PAR-high and PAR-low groups [30]. Neutrophils, the major inflammatory cells, can promote tumor cell proliferation, angiogenesis, and metastasis by inhibiting T cells. Lymphocytes, on the other hand, can prevent tumor progression by enhancing immune surveillance. However, the associated inflammation during tumor development inhibits the lymphocytes, leading to immune escape [31]. Xu et al. indicated that the neutrophil–lymphocyte ratio (NLR) could be a sensitive parameter for evaluating the prognosis in esophageal cancer [32]. Similarly, high pre-treatment NLR was reported to be associated with worse DFS and OS in patients with resectable esophageal cancer [33]. A meta-analysis by Yang et al. also suggested that high NLR is associated with poor prognosis in patients with esophageal cancer [34].

In our study, we found an inverse relationship between the PAR value and overall survival, whereby low PAR values correlated to better survival. Our results did not show any apparent impact of NLR, PLR, nadir lymphocyte count, or serum albumin, on survival. Spleen is the largest lymphoid organ in the body where white pulp activates the immune response when antigens and antibodies are present in blood [35]. An unintended increase in spleen dose can have a significant impact on absolute lymphocyte count and, thereby, tumor immunity. Previous studies had shown a significant correlation between high spleen irradiation doses and low lymphocyte counts after RT in patients with hepatocellular carcinoma and pancreatic cancer [36, 37]. Sakaguchi et al. demonstrated that spleen dose to be associated with decreased lymphocyte count and an increased ratio of NLR after treatment in patients with esophageal cancer [38]. Alexandru et al. suggested that spleen unintentional V15 and maximum dose irradiation were associated with lymphopenia during chemoradiotherapy [39]. However, the present analysis failed to show any statistically significant association between spleen dosimetry (in terms of the V2Gy, V5Gy, V10Gy, V15Gy, V20Gy, the mean, and maximum doses) and lymphopenia.

Our analysis suggested a prognostic implication of the PAR value in patients of esophageal cancer with an inverse association between PAR and OS. Certain limitations of our analysis must be considered. This was a retrospective single-center analysis which may have led to selection bias. Secondly, platelet counts and serum albumin levels could be influenced by other factors such as coagulation disorder and liver dysfunction, which confound the results. Thirdly, the optimal cut-off for PAR and NLR might be different for the Indian population than for the Western population, whose studies have been used to define the cut-off points in this analysis. However, PAR, in the not-so-distant future, may evolve as a novel, convenient, and inexpensive prognostic indicator in esophageal cancer. Future validation from prospective larger-scale studies is warranted.

## Conclusion

This analysis showed that PAR could be a novel and independent predictive variable with survival connotations. Measurement of PAR is a relatively inexpensive, convenient, and reliable endeavor in routine clinical practice. With future studies and robust evidence to show the prognostic importance of PAR, it may become one of the simplest indices that will help in clinical decision-making regarding the intensification of treatment.

## Data Availability

The datasets used and/or analysed during the current study are available from the corresponding author on reasonable request.
